# Hepcidin levels correlate to liver iron content, but not steatohepatitis, in non-alcoholic fatty liver disease

**DOI:** 10.1186/s12876-018-0804-0

**Published:** 2018-06-05

**Authors:** Joel Marmur, Soheir Beshara, Gösta Eggertsen, Liselotte Onelöv, Nils Albiin, Olof Danielsson, Rolf Hultcrantz, Per Stål

**Affiliations:** 1 Unit of Liver Diseases, Department of Upper GI, C1-77 Huddinge, Karolinska University Hospital, Karolinska Institutet, 141 86 Stockholm, Sweden; 2 Unit of Gastroenterology and Hepatology, Department of Medicine, Ersta Hospital, Karolinska Institutet, Stockholm, Sweden; 3 Unit of Clinical Chemistry, Department of Laboratory Medicine, Karolinska University Hospital, Karolinska Institutet, Stockholm, Sweden; 4 Department of Radiology, Ersta Hospital, Karolinska Institutet, Stockholm, Sweden; 5 Unit of Pathology, Department of Laboratory Medicine, Karolinska University Hospital, Karolinska Institutet, Stockholm, Sweden

**Keywords:** Hepcidin, Iron overload, Non-alcoholic fatty liver disease

## Abstract

**Background:**

One-third of patients with non-alcoholic fatty liver disease (NAFLD) develop dysmetabolic iron overload syndrome (DIOS), the pathogenesis of which is unknown. Altered production of the iron-regulatory peptide hepcidin has been reported in NAFLD, but it is unclear if this is related to iron accumulation, lipid status or steatohepatitis.

**Methods:**

Eighty-four patients with liver disease, 54 of which had iron overload, underwent liver biopsy (*n* = 66) and/or magnetic resonance imaging (*n* = 35) for liver iron content determination. Thirty-eight of the patients had NAFLD, 29 had chronic liver disease other than NAFLD, and 17 had untreated genetic hemochromatosis. Serum hepcidin was measured with ELISA in all patients and in 34 controls. Hepcidin antimicrobial peptide (*HAMP*) mRNA in liver tissue was determined with real-time-quantitative PCR in 36 patients.

**Results:**

Serum hepcidin was increased similarly in NAFLD with DIOS as in the other chronic liver diseases with iron overload, except for genetic hemochromatosis. *HAMP* mRNA in liver tissue, and serum hepcidin, both correlated to liver iron content in NAFLD patients (*r*^2^ = 0.45, *p* < 0.05 and *r*^2^ = 0.27, *p* < 0.05 respectively) but not to body mass index, NAFLD activity score or serum lipids. There was a good correlation between *HAMP* mRNA in liver tissue and serum hepcidin (*r*^2^ = 0.39, *p* < 0.01).

**Conclusions:**

In NAFLD with or without dysmetabolic iron overload, serum hepcidin and *HAMP* mRNA in liver correlate to body iron content but not to the degree of steatohepatitis or lipid status. Thus, the dysmetabolic iron overload syndrome seen in NAFLD is not caused by an altered hepcidin synthesis.

## Background

Non-alcoholic fatty liver disease (NAFLD) is the most prevalent liver disease worldwide, with an association to obesity, insulin resistance and the metabolic syndrome [1–3]. Approximately one-third of patients with NAFLD develop elevated serum ferritin and hepatic iron overload, a condition known as the “dysmetabolic iron overload syndrome” (DIOS) [4, 5]. The underlying mechanisms for DIOS are unknown. Increased iron stores could be of pathogenic importance in NAFLD, since it may increase the risk of hepatocyte ballooning, inflammation and fibrosis, which are features of liver damage seen in non-alcoholic steatohepatitis (NASH) which is the more severe form of NAFLD [6–8].

The body’s iron balance is regulated by hepcidin, a 25 amino-acid peptide that inhibits iron uptake in the gut and iron recycling from macrophages, consequently decreasing iron levels in plasma [9]. An inappropriately low hepcidin synthesis has been reported in NAFLD [10, 11] which could facilitate iron uptake and predispose for DIOS, but results are not consistent [12, 13]. Hepcidin levels in NAFLD are difficult to elucidate, since both obesity and diabetes may increase hepcidin production [12, 14, 15]. For example, in morbidly obese subjects, hepcidin is released from adipose tissue [12, 13, 15, 16], which may lead to anemia and entrapment of iron in reticuloendothelial cells [9]. Thus, in NAFLD data is conflicting whether or not hepcidin predominantly correlates to body iron stores [16, 17], to features of the metabolic syndrome [18, 19] or the hepatic inflammation seen in steatohepatitis (NASH). In a recent study, hepatic iron measured by magnetic resonance imaging was found to be the major determinant of serum ferritin in NAFLD [20]. In a large study on individuals with metabolic syndrome, results suggested that that the iron regulatory feedback on hepcidin synthesis was preserved in these patients [21].

The aim of the present study was to elucidate whether body iron stores, steatohepatitis or lipid status in NAFLD correlated to hepcidin synthesis. For this purpose, we compared serum hepcidin levels and hepatic hepcidin antimicrobial peptide (*HAMP*) gene expressions in NAFLD patients with various degrees of iron overload, to those of patients with other forms of acquired or genetic iron overload. We aimed to include patients with various hepcidin levels, and therefore we included untreated hereditary hemochromatosis patients (with a known hepcidin deficiency) as well as patients with iron overload associated to other chronic liver diseases, presumably having elevated serum hepcidin levels. We correlated our findings to iron indices, liver biopsy features, anthropometric data, and lipid parameters.

## Methods

### Patient data collection and investigations

All patients referred to the Unit of Liver Diseases at the Karolinska University Hospital for liver biopsy due to chronic liver disease and/or hemochromatosis, and with an elevated serum ferritin, between January 2008 and April 2013, were asked to participate in the study. Hyperferritinemia was defined as a serum ferritin > 350 μg/L. In addition, patients with chronic liver disease and normal iron parameters undergoing liver biopsy were enrolled for comparison. All patients were over 18 years of age and had given written informed consent. One patient was excluded due to iron deficiency. No patients included had been subject to treatment with iron reduction therapy before entering the study.

A total of 84 patients were enrolled (26 females, 58 males), of which 62 had elevated ferritin levels and 23 a normal serum ferritin concentration. Thirty-eight of the 84 patients had NAFLD, 17 had untreated hereditary hemochromatosis (HH), and 29 had various other causes of chronic liver disease (CLD), such as autoimmune liver disease, alcoholic liver disease, chronic viral hepatitis, alpha-1-antitrypsin deficiency, cryptogenic cirrhosis, porphyria cutanea tarda, methotrexate-induced liver fibrosis, or the hemochromatosis phenotype but without the *C282Y* or *H63D* mutations. All these other etiologies (except NAFLD and *HFE*-associated HH) were thus grouped together as CLD. NAFLD was defined as either Grade 1 or more steatosis in the liver biopsy according to Kleiner et al. [22], or a bright liver with increased echogenicity at abdominal ultrasound investigation. Among the 17 patients with HH, 12 were *HFE* C282Y homozygotes and five were C282Y/H63D compound heterozygotes. In the group of 29 patients with chronic liver disease, eight had a normal iron content in the liver, and 21 had iron overload, and were classified as “chronic liver disease with iron overload” (CLD-IO). One of these had received oral iron substitution for several years; however, none had been treated with parenteral iron substitution or blood transfusions. Amongst the 21 patients classified as CLD-IO, ten had a clinical phenotype of hemochromatosis (elevated serum ferritin and transferrin saturation, and hepatic iron overload) but without homozygosity for the *HFE* C282Y mutation or compound heterozygosity for the *C282Y* and *H63D* mutations, and without alcohol overconsumption. The other 19 CLD patients had alcohol overconsumption (> 30 g/day) (*n* = 9), primary biliary cholangitis (*n* = 2), hepatitis C (*n* = 1), alpha-1-antitrypsin deficiency (*n* = 1), porphyria cutanea tarda (*n* = 1), cryptogenic cirrhosis (*n* = 2), or methotrexate-treated psoriasis (*n* = 3). None of the patients with HH, NAFLD or CLD with the clinical phenotype of hemochromatosis had reported a previous or current alcohol consumption exceeding 20 g/day. Two CLD-IO patients (with alpha-1-antitrypsin deficiency and alcohol overconsumption, respectively) were heterozygous for the *H63D* mutation, and one (with alcohol overconsumption) was heterozygous for the *C282Y* mutation.

Iron overload was defined as a histologic iron score of ≥1 or an estimated iron content > 40 μmol/g on magnetic resonance imaging (MRI) investigation (see below). The patient groups are displayed in Table 1.

**Table 1:** Clinical and laboratory data for patients and controls

	Control *N* = 34	NAFLD *N* = 22	NAFLD with DIOS *N* = 16	CLD *N* = 8	CLD-IO *N* = 21	Compound heterozygous HH *N* = 5	Homozygous HH *N* = 12
Gender (F/M)	19/15	8/14	5/11	4/4	6/15	1/4	2/10
Age (y)	40 ± 10*	54 ± 16	59 ± 10	57 ± 8	58 ± 8	59 ± 9	51 ± 6
BMI (kg/m^2^)	23.3 ± 2.6*	30.4 ± 4.2#	28.1 ± 2.4	27.3 ± 5.1	27.0 ± 4.2	29.0 ± 3.5	28.2 ± 4.8
Hemoglobin (g/L)	142 ± 11	150 ± 15	149 ± 17	140 ± 10	140 ± 17	158 ± 15	154 ± 12
ALT (U/L)	18 ± 6*	94 ± 76	59 ± 41	71 ± 59	53 ± 29	41 ± 35	82 ± 35
CRP (mg/L)	1.1 ± 0.4	3.0 ± 2.9	1.8 ± 0.9	2.8 ± 3.0	3.8 ± 5.5	3.6 ± 4.0	3.3 ± 2.7
Serum ferritin (μg/L)	94 ± 87	304 ± 248	816 ± 285¤	454 ± 688	1304 ± 1295¤	878 ± 408¤	1753 ± 998¤
Transferrin saturation (%)	0.28 ± 0.11	0.28 ± 0.09	0.39 ± 0.09	0.31 ± 0.15	0.50 ± 0.21§	0.46 ± 0.10§	0.76 ± 0.21*
Hepatic iron score	N.D.	0.11 ± 0.21	2.19 ± 0.95¤	0.29 ± 0.27	2.98 ± 1.22¤	3.38 ± 0.75¤	4.35 ± 0.63¤

*CLD* chronic liver disease, *IO* iron overload, *HH* hereditary hemochromatosis, *NAFLD* non-alcoholic fatty liver disease, *DIOS* dysmetabolic iron overload syndrome. Values denote mean ± S.D

*P < 0.05 vs. all other groups

#*p* < 0.05 vs. Control, CLD, CLD-IO

¤*p* < 0.05 vs. Control, NAFLD, CLD

§*p* < 0.05 vs. Control, NAFLD, DIOS, CLD, homozygous HH

Liver biopsy was performed in 66 out of the 84 patients. MRI was used for iron assessment in 35 cases, and in 14 of these, histology was lacking. In 21 cases, there was both histology and MRI. In four cases (two HH homozygotes, one HH compound heterozygote, and one with CLD and normal ferritin) both liver histology and MRI was lacking.

### Data collection from controls

A total of 40 healthy controls, recruited from hospital staff, participated in the study. None had a history of liver disease. Written consent was given. Of the controls, six individuals were excluded (elevated transaminases in one case, compound heterozygosity of the HFE gene and elevated serum ferritin in one case, iron deficiency with serum ferritin < 15 μg/L in three cases, and elevated ferritin (413 μg/L) in one case). The remaining 34 controls were included in the study (Table 1).

Biochemical data was collected at the time of enrollment in the study. Blood samples were drawn before 10 A.M. in the morning. Subjects were not fasting but had had a light breakfast. Routine blood chemistry analyses as well as *HFE* mutation analysis were performed on all subjects at the Department of Clinical Chemistry at Karolinska University Hospital. Body mass index was calculated using the formula: weight in kilogram / (height in meters) ^2^.

### Quantitative assay of hepcidin in serum samples

Freshly drawn samples from the 84 patients and 34 controls were centrifuged and serum was stored at − 70 °C until analysis. Samples were analyzed for hepcidin by a competitive ELISA kit (Bachem, Peninsula Laboratories, LLC, CA, United States) as reported previously [23]. Reference ranges established in 83 normal subjects showed hepcidin levels that ranged 8–76 and 2–50 μg/L for men and women, respectively (2.5–97.5 percentiles). The results were significantly different between genders. As internal controls, pooled sera of 7 and 6 samples representing low (≈0.4 μg/L) and normal (≈3 μg/L) levels respectively were frozen at − 70 °C. Control sera were run in 6 replicates at each assay. The intra-assay variation showed CVs of 18% for low and 13% for normal controls, while inter-assay CVs were 18 and 19%, respectively. The lower limit of detection, calculated as 3 SD above the lowest standard, was 0.05 μg/L and linearity for this kit was determined as between 0,2–5 μg/L (2–50 μg/L for samples diluted 1:10). Samples outside linearity limits were rerun using proper dilution factor, and all samples were run in duplicate.

### Analysis of IL-6 and TNFα

IL-6 and TNFα were measured using Bio-plex Pro Human Cytokine Group 1 kit (Bio-rad Laboratories, Hercules, CA, USA) according to the manufacturer’s instructions. Briefly, plasma/serum was diluted 1:4 using Bio-plex sample diluents. To obtain the nine point (including blank) standard curve, the kit standard was reconstituted and diluted fourfold. The 10× IL-6 and TNFα coupled beads were diluted in kit assay buffer and added to all standard and sample wells. The plate was incubated on shaker 30 min. After washing IL-6 and TNFα biotinylated detection antibodies were added and the plate was incubated as above. In the final step, PE-conjugated Streptavidin was added and the plate was run on a Magpix instrument (Luminex Corporation, Austin TX, USA) and analyzed with xPonent software (Luminex).

### Analysis of HAMP mRNA in liver biopsies

Sixty-six patients underwent liver biopsy, and tissue from 39 of these was collected for hepcidin mRNA analysis. Tissues were immediately immersed in RNAlater and stored at − 70°C until processed. Total RNA was successfully retrieved from 36 of the 39 utilized liver biopsies with a dry weight of 0.3–5.9 mg using the RNAqueous -4PCR kit (Ambion PN AM1914). Recovered quantities of RNA ranged from 13 to 200 ng/μL. The quality and quantity of the extracted RNA was verified with the Bio-Rad Experion 700–7000 electrophoresis system, and only samples with an RQI > 8 were included in the study. cDNA synthesis was carried out with the High Capacity Reverse Transcriptase Kit (Applied Biosystems), using 65–930 ng of total RNA per sample. Determination of specific mRNA levels was performed as described previously [23].

### Histologic examination of liver biopsy samples

Liver biopsy samples were revalued by an experienced pathologist (O.D.) blinded to clinical data. Samples from NAFLD-patients were evaluated for the degree of steatosis (0–3), lobular inflammation (0–3) and hepatocellular ballooning (0–2) according to Kleiner et al. [22]. The unweighted sum of these three variables were used to calculate the NAFLD activity score (NAS). Patients with NAS ≥5 were diagnosed with NASH.

Siderosis was determined for all patients semi-quantitatively on histopathologic examination of Perls’ stained liver biopsy samples adapted from Deugnier et al. [24] to match available levels of magnification.

An iron score from 0 to 4 for iron in hepatocytes was determined as follows: [0] granules absent or barely discernible at a magnification of 400X; [1] barely discernible granules at a magnification of 200X but easily confirmed at a magnification of 400X; [2] discrete granules at 100X magnification; [3] discrete granules easily confirmed at magnification of 40X, but barely discernible at a magnification of 20X; [4] granules obvious at a magnification of 20X, and barely visible for the naked eye. RES-iron was also determined and scored as [0] none, [1] mild, [2] or more than mild, as described by Nelson et al. [25]. These two scores were transformed into a histologic iron score (HIS) ranging from 0 to 5, comprising the score for iron in hepatocytes (0–4), plus one point for RES iron in those cases where it had been determined as more than mild, or a half point where it has been determined as mild. Iron overload was defined as a histologic iron score of ≥1.

### Magnetic resonance imaging (MRI)

**Fig. 1:**
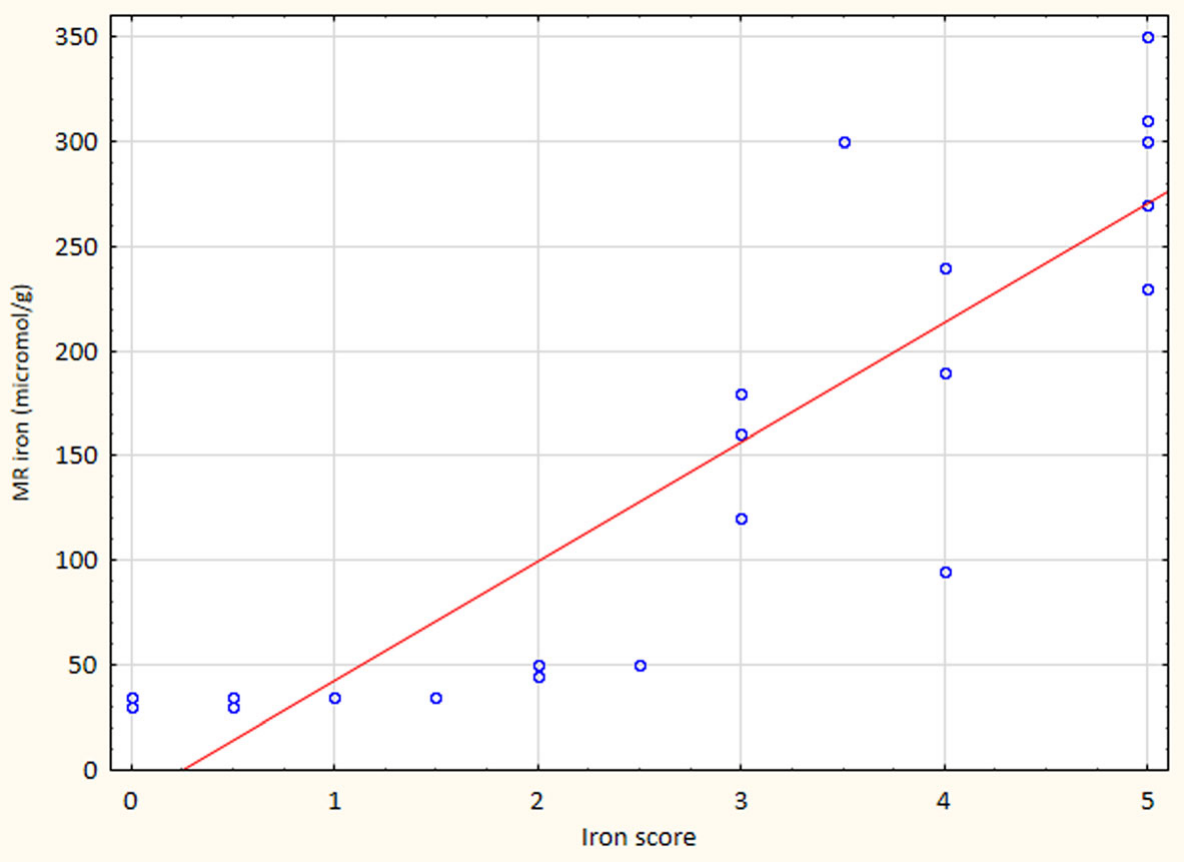
Graph demonstrating the correlation between MRI iron content (μmol/g) and histological iron score in 21 patients in whom both MRI and liver biopsy was performed. There was a good correlation between these variables (*r*^2^ = 0.77; *p* < 0.01)

MRI was used for detection and quantification of liver iron overload in 35 patients and correlated to histology in 21 of these (Fig. 1). The liver iron was assessed semi-quantitatively as described by Gandon et al. [26].

In the correlation analyses of serum hepcidin to liver iron content, MRI iron was approximated to histologic liver iron (HIS) score based on the correlation estimated from Fig. 1: < 40 μmol iron/g tissue  = HIS 0; 40–74 μmol/g = HIS 1; 75–129 μmol/g = HIS 2; 130–179 μmol/g = HIS 3; 180–239 μmol/g = HIS 4; ≥240 μmol iron/g tissue = HIS 5.

### Statistical analyses

The relationship between two categorical variables was examined with Chi^2^-test or Fisher’s exact test (when applicable). Numerical values of laboratory parameters were analyzed using one-way ANOVA and validated for equal variance and normal distribution. Kruskal-Wallis ANOVA was used when the assumptions did not hold. The correlation between two numerical variables was analyzed with simple linear regression validated for linearity, variance between observations and for normal distribution. In the cases where the assumptions did not hold the Spearman’s rank order correlation was used instead. Multiple linear regression was used for variables that were significantly correlated to serum hepcidin in the simple linear regression. A *p*-value < 0.05 was considered statistically significant.

## Results

### Clinical and laboratory data

The distribution of patients, and clinical and laboratory data of patients and controls are demonstrated in Table 1. Controls were significantly younger than patients, and had lower BMI, ALT and serum ferritin levels. BMI was highest in the NAFLD patient group. Transferrin saturation was significantly increased in patients with homozygous HH, and in the 10 CLD-IO patients with a HH phenotype without HFE mutations, compared with the other patient groups and controls. Hepatic iron score did not differ significantly between patients with DIOS and CLD-IO.

### Distribution of HFE mutations

The distribution of *HFE* mutations are shown in Table 2. Among patients with chronic liver disease and iron overload (CLD-IO), four were heterozygous for C282Y, two homozygous and one heterozygous for H63D. The H63D mutation was significantly more frequent in patients with NAFLD as compared with the controls (*p* < 0.05).

**Table 2:** *HFE* genotypes in patients and controls

	wt/wt	C282Y/wt	C282Y/C282Y	C282Y/H63D	H63D/wt	H63D/H63D
Controls (*n* = 34)	26	4	–	–	4	–
NAFLD with normal iron stores (*n* = 22)^a^	13	1	–	–	6*	1
NAFLD with DIOS (*n* = 16)	12	2	–	–	2	–
CLD (*n* = 8)^b^	4	–	–	–	2	–
CLD-IO (*n* = 21)	14	4	–	–	1	2
Compound heterozygous HH (*n* = 5)	–	–	–	5	–	–
Homozygous HH (*n* = 12)	–	–	12	–	–	–

*CLD* chronic liver disease, *IO* iron overload, *HH* hereditary hemochromatosis, *NAFLD* non-alcoholic fatty liver disease, *DIOS* dysmetabolic iron overload

*p < 0.05 in patients with NAFLD vs. controls

^a^one missing value

^b^two missing values

### Correlation analysis of histologic iron score and hepatic iron content determined by MRI

Simple linear regression showed a good correlation between histologic iron score and hepatic iron content determined by MRI, as demonstrated in Fig. 1 (*r*^2^ = 0.77, *p* < 0.01).

### Serum hepcidin and hepcidin mRNA in liver biopsies

**Fig. 2:**
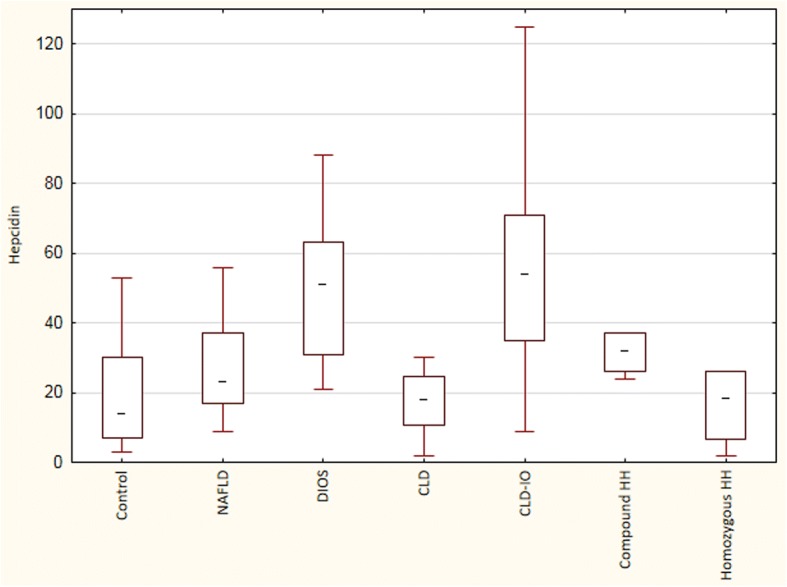
Serum hepcidin levels (μg/L) in the different patient groups. The box plots show the median, the interquartile range and the min-max values. Hepcidin levels were significantly increased in chronic liver disease with iron overload (CLD-IO) and non-alcoholic fatty liver disease with dysmetabolic iron overload (NAFLD-DIOS) compared with the other groups (Kruskal-Wallis ANOVA, *p* < 0.05)

**Fig. 3:**
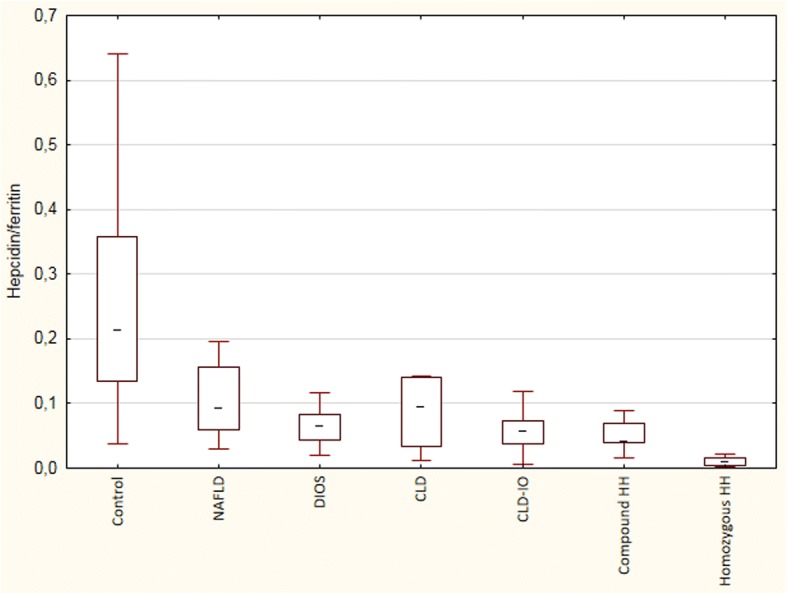
The ratios between serum hepcidin (μg/L) and serum ferritin (μg/L). Patients with homozygous HH had significantly lower ratios compared with the other groups (Kruskal-Wallis ANOVA, *p* < 0.05)

Serum hepcidin values for the different patient groups and controls are shown in Fig. 2. Serum hepcidin levels were significantly increased in patients with NAFLD with DIOS and in those with chronic liver disease with iron overload (CLD-IO) compared with the other groups. The ratios between serum hepcidin and ferritin are shown in Fig. 3. As expected, this ratio was significantly reduced in homozygous HH compared with the other groups. Among patients with CLD-IO, this ratio was slightly lower in those with alcoholic liver disease (ALD) or hepatitis C (0.049±0.034) as compared with those without alcohol overconsumption (0.058±0.032), or DIOS patients (0.070±0.037), although these differences were not statistically significant.

**Fig. 4:**
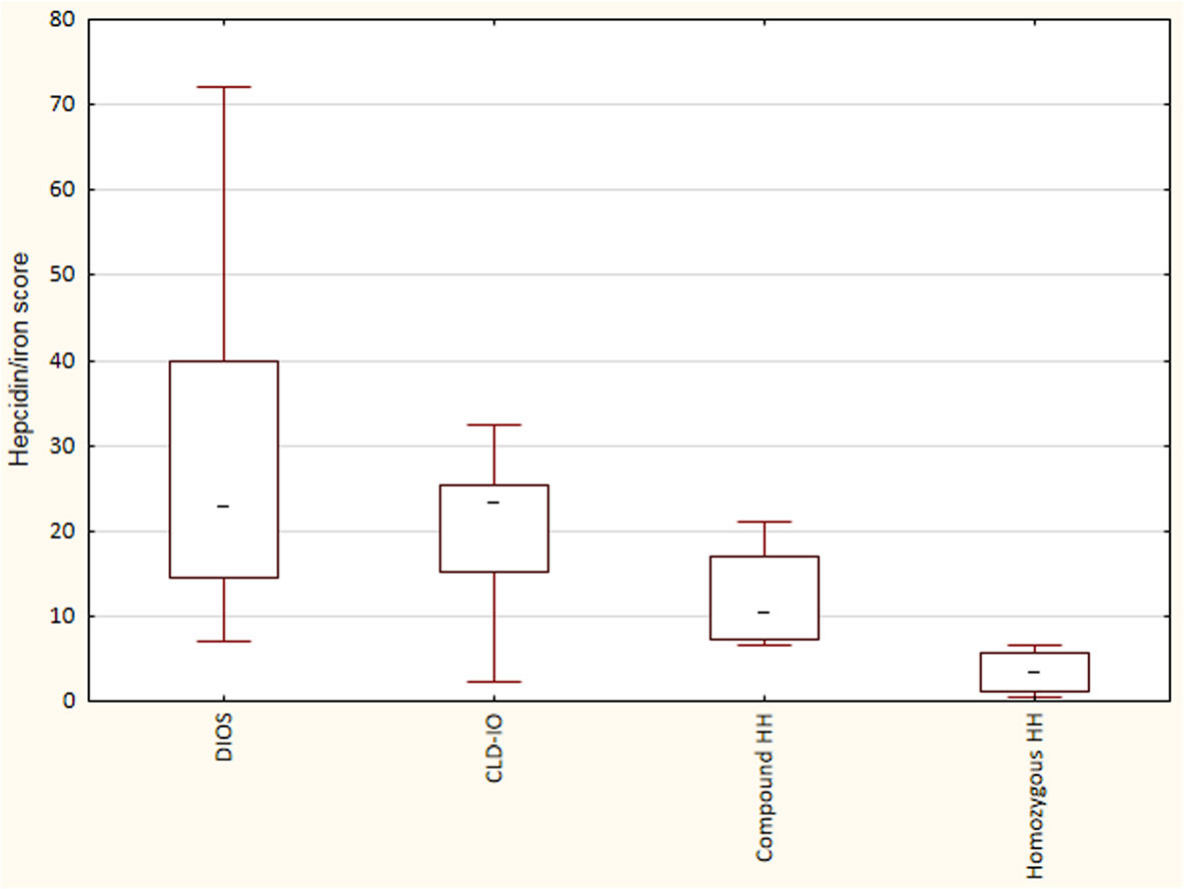
The ratios between serum hepcidin (μg/L) and hepatic iron contents (“iron score”). The calculation of iron scores is described in Methods. Patients with homozygous HH had significantly lower ratios compared with the other groups (Kruskal-Wallis ANOVA, *p* < 0.05)

Figure 4 shows the ratios between serum hepcidin and hepatic iron score, which was similar in patients with CLD-IO and DIOS, and reduced in those with homozygous HH. The hepcidin/iron score ratio was slightly lower in those with ALD or hepatitis C (18.7±8.1) as compared with those without alcohol overconsumption (22.4±10.2), or DIOS (30.8±23.7), however not statistically significant. There was a significant correlation between serum hepcidin levels and hepatic *HAMP* mRNA (*r*^2^ = 0.39, *p* < 0.01).

### Clinical, laboratory and histological findings in patients with NAFLD with or without DIOS (Table 3)

Serum hepcidin, serum transferrin saturation and hepatic iron score were all significantly higher in NAFLD with DIOS as compared with NAFLD without DIOS (*p* < 0.05). Serum levels of triglycerides or total cholesterol did not differ significantly between the groups. Levels of TNF-α and IL-6 were highest in NAFLD without DIOS and  elevated serum ferritin (difference not statistically significant). *HAMP* mRNA in liver tissue correlated to the hepatic iron score (*r*^2^ = 0.45, *p* < 0.05) but not to NAFLD activity score (*r*^2^ = 0.003, *p* < 0.89). Serum hepcidin correlated significantly to serum ferritin (*r*^2^ = 0.20, *p* < 0.01) and serum transferrin saturation (*r*^2^ = 0.17, *p* < 0.01) but not to BMI, TNF-α, IL-6, triglycerides or cholesterol. In multiple linear regression analysis only ferritin correlated significantly to serum hepcidin levels when adjusted for other variables. There was no significant difference in stage of fibrosis, grade of steatosis, ballooning, lobular inflammation or NAFLD activity score between the groups (Table 3).

**Table 3:** Clinical, laboratory and liver biopsy findings in patients with NAFLD with and without dysmetabolic iron overload syndrome (DIOS), and normal vs. elevated serum ferritin, respectively

	NAFLD without DIOS (*n* = 22)		NAFLD with DIOS (*n* = 16)
	With serum ferritin < 350 μg/L (*n* = 15)	With serum ferritin > 350 μg/L (*n* = 7)	
Serum hepcidin (μg/L)	24 ± 19	37 ± 13	53 ± 28*
Serum ferritin (μg/L)	156 ± 78*	621 ± 170	816 ± 285
Transferrin saturation (%)	28 ± 8	25 ± 10	39 ± 9#
Liver iron score	0.03 ± 0.13	0.14 ± 0.24	2.13 ± 0.92*
CRP (mg/L)	2.7 ± 2.2	3.7 ± 4.2	1.8 ± 0.91
Plasma-triglycerides (mmol/L)	2.89 ± 1.09	1.83 ± 1.09	1.95 ± 0.90
Plasma cholesterol (mmol/L)	5.18 ± 0.96	5.25 ± 0.84	5.25 ± 0.71
TNF-α (ng/L)	6.27 ± 5.13	137 ± 316	8.37 ± 5.82
IL-6 (ng/L)	2.47 ± 1.71	32.3 ± 62.7	2.36 ± 1.02
NAS	4.5 ± 1.8	3.6 ± 1.7	4.4 ± 1.8
Steatosis (grade)	2.07 ± 0.80	2.00 ± 1.00	2.72 ± 0.65
Ballooning	1.07 ± 0.59	0.80 ± 0.84	0.63 ± 0.67
Lobular inflammation	1.20 ± 0.86	1.00 ± 0.71	1.00 ± 1.00
Portal inflammation	0.27 ± 0.46	0.60 ± 0.55	0.18 ± 0.40
Fibrosis	1.33 ± 0.90	2.00 ± 1.00	2.72 ± 0.65

Values denote mean ± S.D

*=*p* < 0.05 (vs. the other groups)

# = *p* < 0.05 (vs. NAFLD with serum ferritin > 350 μg/L)

## Discussion

In the present study, we demonstrate that in NAFLD patients, hepcidin in serum and *HAMP* mRNA in liver tissue correlate significantly to body iron stores, regardless if they are expressed as serum ferritin or liver iron content. Furthermore, there was no correlation to the degree of steatohepatitis (defined as NAFLD activity score), to lipid parameters (serum cholesterol or triglycerides), body mass index, or C-reactive protein. We found that serum hepcidin levels in NAFLD patients with dysmetabolic iron overload (DIOS) are similar to those found in other liver diseases with iron overload (CLD-IO), except for hereditary hemochromatosis, in which patients have an inherited hepcidin deficiency. In our patient cohort without morbid obesity, hepatic *HAMP* mRNA levels showed a good correlation to the serum hepcidin values measured by ELISA. When calculating the hepcidin levels in relation to serum ferritin (Fig. 3) or to the liver iron score (Fig. 4), patients with DIOS had overall similar ratios as patients with CLD-IO, although those with alcoholic liver disease and hepatitis C had a trend to somewhat lower levels. Others have demonstrated that hepatic iron is the major determinant of serum ferritin levels in NAFLD, results in line with the present study [20]. Together, these findings point at an adequate hepcidin synthesis in NAFLD in relation to iron stores, and the iron accumulation in DIOS cannot be explained by hepcidin deficiency, in contrast to what is seen in hereditary hemochromatosis.

Some other previous studies have presented conflicting results. Barisani et al. found an inadequate hepcidin production for a given level of iron status in NAFLD patients compared to controls, although not as low as in beta-thalassemia or hereditary hemochromatosis [11]. In contrast, several other studies found hepcidin levels to correlate to iron parameters in NAFLD and DIOS [17, 21, 27, 28]. Senates et al. found an association between serum hepcidin and cholesterol and triglycerides levels, but not with iron parameters [18], which contrasts with our findings. In obesity, hepcidin can be produced by adipose tissue [13, 28], possibly through activation of hemojuvelin gene expression [29]. Thus, in morbidly obese patients undergoing bariatric surgery, hepcidin levels correlate to the grade of obesity, but not to the degree of fat in the liver tissue [12, 15]. Likewise, the presence of NASH did not alter the expression of *HAMP* mRNA in adipose tissue [13]. Low-grade inflammation associated with obesity could lead to elevation of both serum ferritin and hepcidin levels. In inflammatory conditions, elevated serum hepcidin would diminish iron uptake and mobilization, possibly causing entrapment of iron in Kupffer cells [19]. However, none of our patients were morbidly obese, and the strong correlation between hepcidin in serum and *HAMP* mRNA in liver tissue in the present study indicates a negligible contribution from adipose tissue to hepcidin synthesis in our cohort.

It has been reported that hepcidin levels were depressed in patients with chronic hepatitis C [30] or alcoholic liver disease [31], suggesting that hepcidin deficiency play a role in the iron accumulation seen in these conditions. As compared to NAFLD-DIOS in our cohort, we found a somewhat lower hepcidin-to-ferritin and hepcidin-to-iron score ratios in patients with alcoholic liver disease and hepatitis C, although the present study was underpowered to detect a true difference in this regard. This finding is in line with the view that there is an adequate hepcidin synthesis in NAFLD-DIOS, why other explanations for the iron accumulation in this condition have to be sought for [16].

We did not find an increased frequency of *C282Y* or *H63D* mutations in NAFLD patients with DIOS as compared to patients with other liver diseases, or healthy controls. However, the *H63D* mutation was enriched in NAFLD patients with normal iron stores, indicating that this mutation may play a role in NAFLD pathogenesis, as suggested previously [17].

Eighteen of our 84 patients did not agree to undergo liver biopsy. In these cases, iron assessment was instead performed by magnetic resonance imaging (MRI), which is considered to be an accurate method to quantify iron overload in the range 60–375 μmol/g [32]. It is not influenced by steatosis or fibrosis and in patients with cirrhosis it may be even more accurate than biopsy [26]. In 21 cases, we performed both liver biopsy and MRI, obtaining a good correlation in cases with a hepatic iron score of 2 or more.

The strength of the present study is that hepcidin was measured both in serum and as *HAMP* mRNA in liver tissue, the correlations of which were excellent. Furthermore, iron content was assessed both with MRI and liver biopsy, and NAFLD patients were compared with other patient cohorts with various degree of iron overload, including genetic hemochromatosis who has an inherited hepcidin deficiency. The major limitation of the study is the small cohort, making it underpowered to perform sub-analyses of various patient groups, e.g. NAFLD without DIOS, alcoholic liver disease or hepatitis C. Also, the smaller size and the younger age of the control group in the present study, relative to the patient cohort, is a limitation when comparing serum hepcidin levels in liver disease patients and healthy controls.

Future studies need to focus on hepcidin-independent mechanisms for the iron-loading seen in NAFLD with DIOS. Hitherto published data indicate that activated iron regulatory protein-1 and increased expression of duodenal divalent metal transporter-1 have been found in NASH [33]. Also, bone morphogenic protein-binding endothelial regulator [34] and hepatocyte nuclear factor-4alpha [35] have been reported to influence iron absorption, and in mice, a high fat diet by itself could increase iron absorption [36]. An impairment in the ability of hepcidin to inhibit iron absorption was demonstrated in DIOS, suggesting hepcidin resistance in this condition [37]. Nevertheless, it is unknown if the iron loading seen in up to one-third of patients with NAFLD is a consequence of the altered lipid metabolism, or an altered expression of iron-regulatory genes, or a combination of both. This topic warrants future research.

## Conclusions

In conclusion, we found that in patients with non-alcoholic fatty liver disease with or without dysmetabolic iron overload, serum hepcidin correlates to iron indices such as serum ferritin, transferrin saturation and liver iron contents, but not to body mass index, NAFLD activity score, or lipid parameters. Hepcidin levels in NAFLD-DIOS are similar to those found in other liver diseases with iron overload, except for genetic hemochromatosis. These data indicate that NAFLD-DIOS is a condition with an adequate hepcidin synthesis and preserved iron-regulatory feedback.

## Abbreviations

ALD: Alcoholic liver disease
CLD: Chronic liver disease
CLD-IO: Chronic liver disease with iron overload
DIOS: Dysmetabolic iron overload syndrome
HAMP: Hepcidin antimicrobial peptide
HH: Hereditary hemochromatosis
MRI: Magnetic resonance imaging
NAFLD: Non-alcoholic fatty liver disease
NASH: Non-alcoholic steatohepatitis
